# Tracking Single Cells in Live Animals Using a Photoconvertible Near-Infrared Cell Membrane Label

**DOI:** 10.1371/journal.pone.0069257

**Published:** 2013-08-26

**Authors:** Alicia L. Carlson, Joji Fujisaki, Juwell Wu, Judith M. Runnels, Raphaël Turcotte, Cristina Lo Celso, David T. Scadden, Terry B. Strom, Charles P. Lin

**Affiliations:** 1 Center for Systems Biology and Wellman Center for Photomedicine, Massachusetts General Hospital and Harvard Medical School, Boston, Massachusetts, United States of America; 2 Department of Biomedical Engineering, Boston University, Boston, Massachusetts, United States of America; 3 Center for Regenerative Medicine, Massachusetts General Hospital, Boston, Massachusetts, United States of America; 4 Cancer Center, Massachusetts General Hospital, Boston, Massachusetts, United States of America; 5 Harvard Stem Cell Institute, Cambridge, Massachusetts, United States of America; 6 Department of Stem Cell and Regenerative Biology, Harvard University, Cambridge, Massachusetts, United States of America; 7 Transplant Institute, Beth Israel Deaconess Medical Center and Harvard Medical School, Boston, Massachusetts, United States of America; University of Frankfurt - University Hospital Frankfurt, Germany

## Abstract

We describe a novel photoconversion technique to track individual cells *in vivo* using a commercial lipophilic membrane dye, DiR. We show that DiR exhibits a permanent fluorescence emission shift (photoconversion) after light exposure and does not reacquire the original color over time. Ratiometric imaging can be used to distinguish photoconverted from non-converted cells with high sensitivity. Combining the use of this photoconvertible dye with intravital microscopy, we tracked the division of individual hematopoietic stem/progenitor cells within the calvarium bone marrow of live mice. We also studied the peripheral differentiation of individual T cells by tracking the gain or loss of FoxP3-GFP expression, a marker of the immune suppressive function of CD4^+^ T cells. With the near-infrared photoconvertible membrane dye, the entire visible spectral range is available for simultaneous use with other fluorescent proteins to monitor gene expression or to trace cell lineage commitment *in vivo* with high spatial and temporal resolution.

## Introduction

Understanding of biological processes would be enhanced and cell-based therapies improved by knowing the exact *in vivo* location and environmental factors that regulate cell division and differentiation. However, conventional population-based tracking techniques have left many critical questions unresolved. For example, it has been difficult to determine where individual stem cells actually divide and differentiate *in vivo*, despite extensive research focused on identifying the microenvironment that regulates stem cell fate [1]–[4]. In conventional studies of cell lineage and development, heterogeneities and possible contaminants in the population of interest have led to ambiguities in the interpretation of the results [5]–[9].

It is therefore necessary to develop a method to track single-cell division and differentiation *in vivo*, which will uncover cellular dynamics that are lost in the ensemble averaging of traditional population-based studies. Elegant *in vitro* demonstrations of stem cell lineage commitment by time-lapse imaging have been described [10], [11] and studies of cell movement and cell-cell interactions in live animals have become possible with the development of confocal and multiphoton intravital microscopy (IVM) [12]–[16]. *In vivo* studies of cell division and differentiation, however, are limited by the length of time the animal can be kept under anesthesia (hours). Alternatively, images can be acquired over multiple imaging sessions, provided that a method exists to locate the same field of view when the animal is repositioned on the stage [3], [17]. This approach can greatly extend the recording time span, but the missing time gaps between imaging sessions can translate into knowledge gaps (e.g., the target cell can move out of the field of view or other cells can move in and be mistaken as the original cell or its progeny) unless additional measures are taken to mark the cells of interest to ensure that the same cells are being tracked with no mistaken identity.

One way to highlight the cells of interest *in vivo* for subsequent tracking is to use photoswitchable [18]–[28] or photoconvertible [18], [29]–[40] fluorescent proteins. However, fluorescent proteins have distinct disadvantages that limit their use for tracking cell division *in vivo* over long time periods. Firstly, loading fluorescent proteins into cells requires transfection, which can change the phenotype of some target cells. Secondly, after photoconversion, new fluorescent proteins produced by the cell will express the original color. Therefore, significant loss of the photoconverted signal will occur through protein turnover and the photoconverted cells revert back to their original color within 24 hours of photoconversion [37], [41]. Finally, although transgenic mice expressing photoconvertible fluorescent proteins exist [36], [37], in order to visualize cell differentiation, new transgenic mice in which the target cells express the photoconvertible fluorescent proteins together with a second fluorescent proteins that marks the differentiation status or function of the cell will have to be generated.

**Figure 1:**
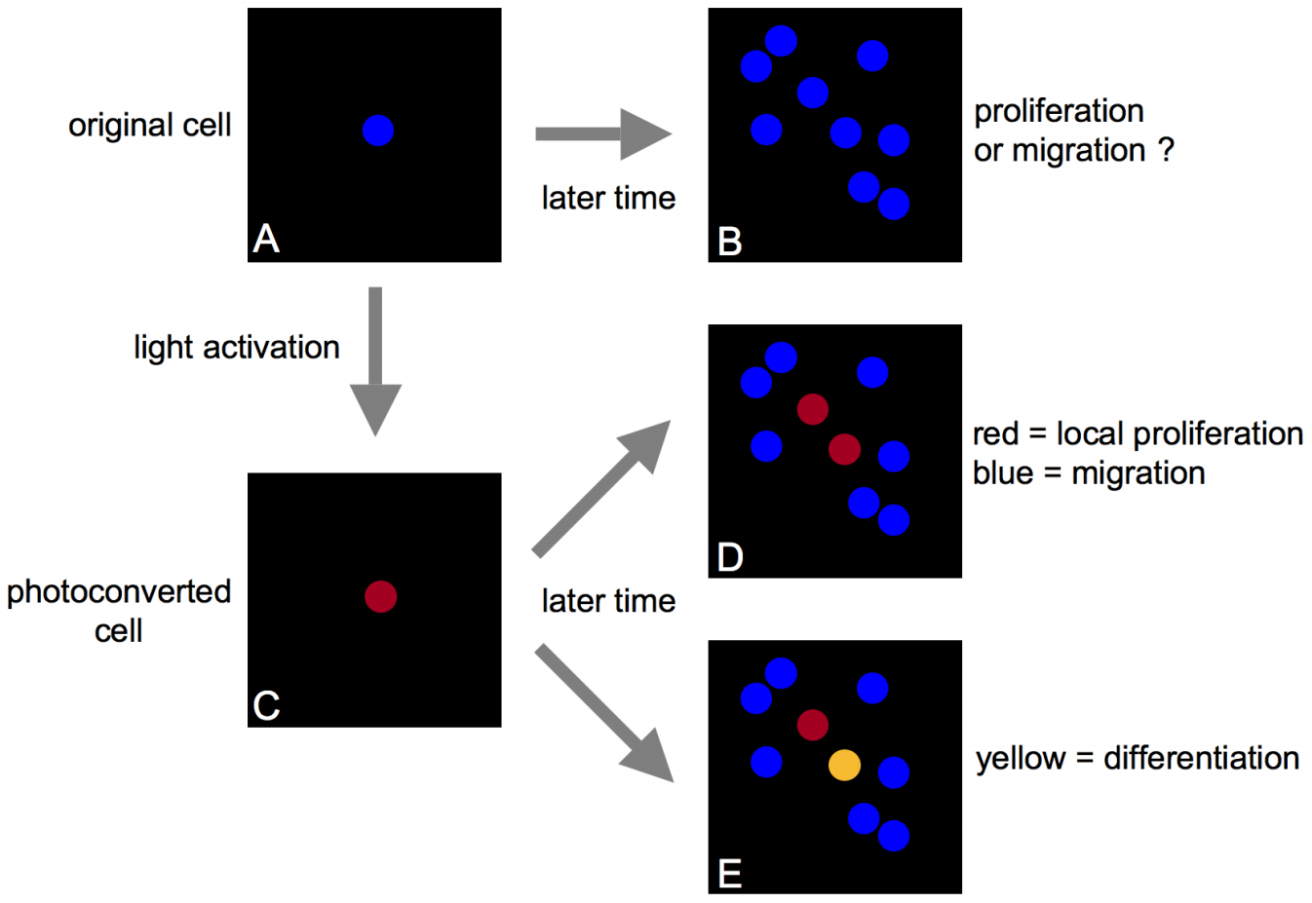
Schematic drawing of *in vivo* photoconversion method to track fate of a single cell. (A) One DiR-labeled cell (blue circle) in the field of view. (B) Without photoconversion, when the same area is imaged at a later time point, additional DiR-labeled cells may be found in the same area, such that proliferation of the single cell viewed previously cannot be distinguished from new cell infiltration. (C) Photoconversion of the DiR-labeled cell (A, blue circle) changes its fluorescence emission (C, red circle), highlighting that cell so that it can be followed longitudinally to track its fate, including both cell division (D) as well differentiation when utilizing a fluorescent reporter gene to mark cell differentiation or function (E). During cell division, the progeny will retain the photoconverted fluorescence color (single red cell in (C) becomes two red cells in (D) through cell division). During differentiation, a photoconverted cell will change its fluorescence color when a reporter gene is turned on or off (red cell in (E) becomes yellow when GFP reporter is turned on).

We have, instead, developed a simple photoconversion technique for long-term tracking of single-cell division and differentiation *in vivo* using a commercial lipophilic membrane dye, DiR (DiIC_18_(7); Invitrogen, Carlsbad, CA). DiR can be used to label cells, including freshly isolated cells, with no known effect on their homing or proliferation [3]. The dye exhibits a permanent change in the fluorescence emission spectrum after photoconversion, and ratiometric imaging can be used to distinguish photoconverted and non-photoconverted cells with high sensitivity. The ratio for each cell remains stable with no reversion to the original color. A schematic drawing depicting the concept of cell tracking by photoconversion is shown in Fig. 1. In this drawing, one DiR-labeled cell seen at an initial time point (Fig. 1A) cannot be distinguished among multiple cells at the same location at a later time point (Fig. 1B). Therefore, proliferation of the initial cell of interest (Fig. 1A) cannot be distinguished from new cell infiltration with certainty. Using light activation to induce photoconversion, the fluorescence emission of only the cell of interest can be changed (Fig. 1C), highlighting that cell so that it can be followed longitudinally to track its fate, including both cell division (Fig. 1D) as well differentiation (when marked by a genetically encoded fluorescent reporter) (Fig. 1E). During cell division, the progeny will retain the photoconverted fluorescence color (Fig. 1D). During differentiation, a photoconverted cell will change its fluorescence color when a reporter gene is turned on or off (Fig. 1E).

Using a custom IVM system and the photoconversion technique, we tracked the division of individual hematopoietic stem/progenitor cells (HSPCs) and mature T cells in the calvarium bone marrow (BM) of live mice for up to 135 h. We have further tracked the peripheral differentiation of DiR-labeled T cells and regulatory T cells by monitoring the gain or loss of the expression of green fluorescence protein (GFP) co-expressed with FoxP3, a transcription factor that governs the immune suppressive function of CD4^+^ T cells [42]–[44]. These data show that optically marking cells by the DiR dye-photoconversion technique enables a single cell or population of cells to be tracked over long time periods *in vivo*.

## Methods

### Ethics statement

Animal studies were conducted under the approval and guidelines of the Massachusetts General Hospital Subcommittee on Research Animal Care. Animals were maintained in accordance with the “Guide for the Care and Use of Laboratory Animals” (National Research Council, 1996). Massachusetts General Hospital (MGH) is registered with the U.S. Department of Agriculture Animal and Plant Health Inspection Service (Certificate No. 14-R-0014) and the Massachusetts Department of Public Health (License No. 11-0022) as a licensed animal research facility. MGH files an annual Letter of Assurance (File No. A3596-01) with the NIH Office of Laboratory Animal Welfare confirming compliance with PHS regulations pertaining to laboratory animal care and use. In addition, the hospital is accredited by the Association for the Assessment and Accreditation of Laboratory Animal Care International (AAALAC). All procedures were performed on sedated animals under Ketamine/Xylazine anesthesia (100 mg/kg Ketamine+15 mg/kg Xylazine), appropriate analgesia was administered as needed, and all efforts were made to minimize suffering.

### Animals

FoxP3 GFP knock-in mice (C57BL/6 background) and C57BL/6 mice were purchased from Jackson Laboratory (Bar Harbor, ME). RAG2^−/−^ mice were purchased from Taconic (Hudson, NY).

### HSPC isolation

Whole bone marrow was obtained from C57BL/6 mice by crushing the femur, tibia, iliac, humerus, and vertebral bones. Cells were pooled, washed, and incubated in a lineage cocktail consisting of biotinylated B220, Mac1, GR-1, CD3a, CD8a, CD4 and Ter119 antibodies (BD Bioscience, Franklin Lakes, NJ). After washing, cells were incubated with MACS-SA beads (Miltenyi, Invitrogen, Carlsbad, CA) per manufacturer's protocol, washed again, and then separated on an LD depletion column in a MidiMACS separation unit in order to remove lineage-specific cell populations. Lineage-negative cells were then stained with Ckit (eBioscience, San Diego, CA) and Sca1 antibodies (BD Bioscience, Franklin Lakes, NJ). Subsequently, Sca1+Ckit+Lin- cells were isolated using a FACS Aria (Becton Dickinson, Franklin Lakes, NJ).

### T cell isolation

Spleen and peripheral cervical, axillary, and inguinal lymph nodes were removed from C57BL/6 FoxP3-GFP mice and cells were harvested by disruption and aspiration of the tissues. Cells were centrifuged and re-suspended in media prior to ACK lysis of the red blood cells. After staining with anti-CD4 antibody (BD Bioscience, Franklin Lakes, NJ), CD4+GFP− cells or CD4+GFP+ cells were isolated using a FACS Aria (Becton Dickinson, Franklin Lakes, NJ).

### DiR cell labeling

**Figure 2:**
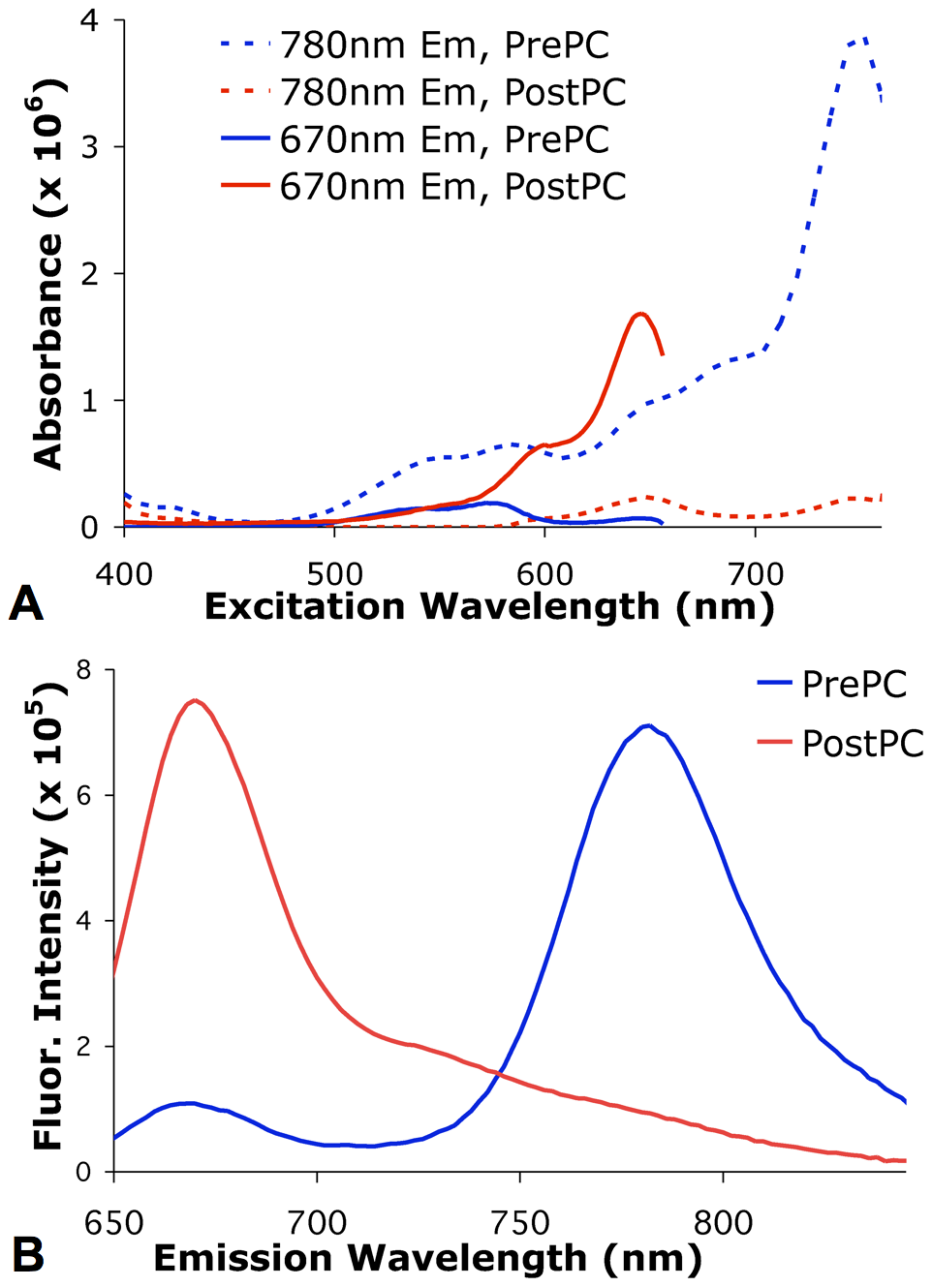
DiR dye spectra before and after photoconversion. (A) Fluorescence excitation spectra of DiR-labeled cells before (PrePC) and after (PostPC) photoconversion acquired at 670 nm and 780 nm emission. (B) Fluorescence emission spectra of DiR-labeled cells before (PrePC) and after (PostPC) photoconversion when excited at 632 nm, showing a significant shift in the fluorescence peak from 780 nm to 670 nm following photoconversion.

**Figure 3:**
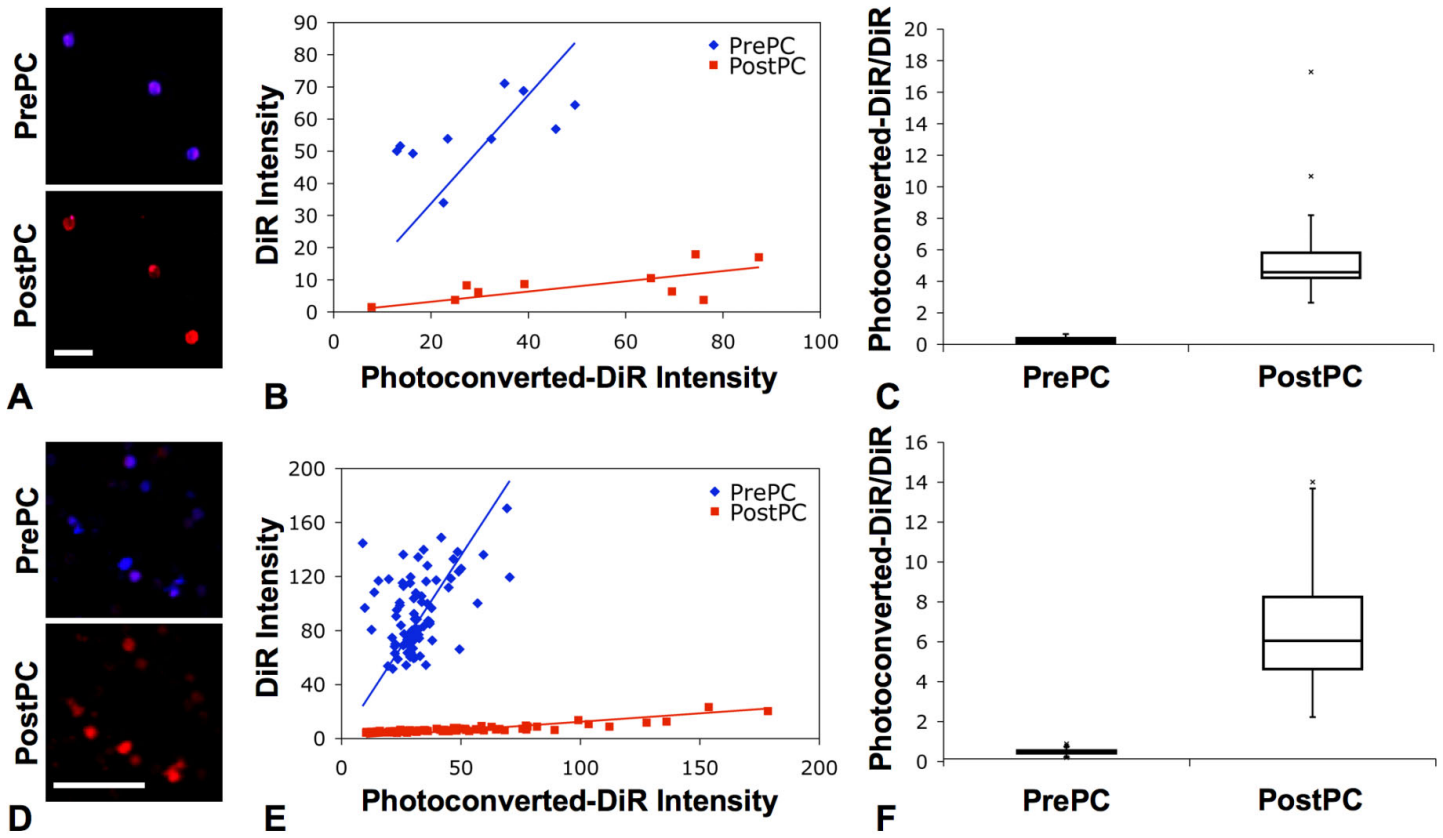
*Ex vivo* photoconversion of DiR-labeled cells. (A) Fluorescence confocal images of *ex vivo* DiR-labeled HSPCs acquired before (PrePC) and after (PostPC) photoconversion (blue: DiR, 760–810 nm; red: photoconverted-DiR, 660–760 nm). Scale bar: 50 µm. (B) Plot of fluorescence intensity of *ex vivo* HSPCs before (PrePC) and after (PostPC) photoconversion for each individual cell. (C) Boxplot of the ratios of the photoconverted-DiR intensity to the DiR intensity, showing ability to photoconvert DiR-labeled stem and progenitor cells and to distinguish the change in the fluorescence intensity ratio after photoconversion (p = 8.36×10^−4^). (D) Fluorescence confocal images of *ex vivo* DiR-labeled T cells acquired before (PrePC) and after (PostPC) photoconversion (blue: DiR, >770 nm; red: photoconverted-DiR, 670–720 nm). Scale bar: 50 µm. (E) Plot of fluorescence intensity of *ex vivo* T cells before (PrePC) and after (PostPC) photoconversion. (F) Boxplot of the fluorescence intensity ratios, also showing ability to photoconvert DiR-labeled T cells and to distinguish the change in fluorescence after photoconversion (p = 1.15×10^−33^).

**Figure 4:**
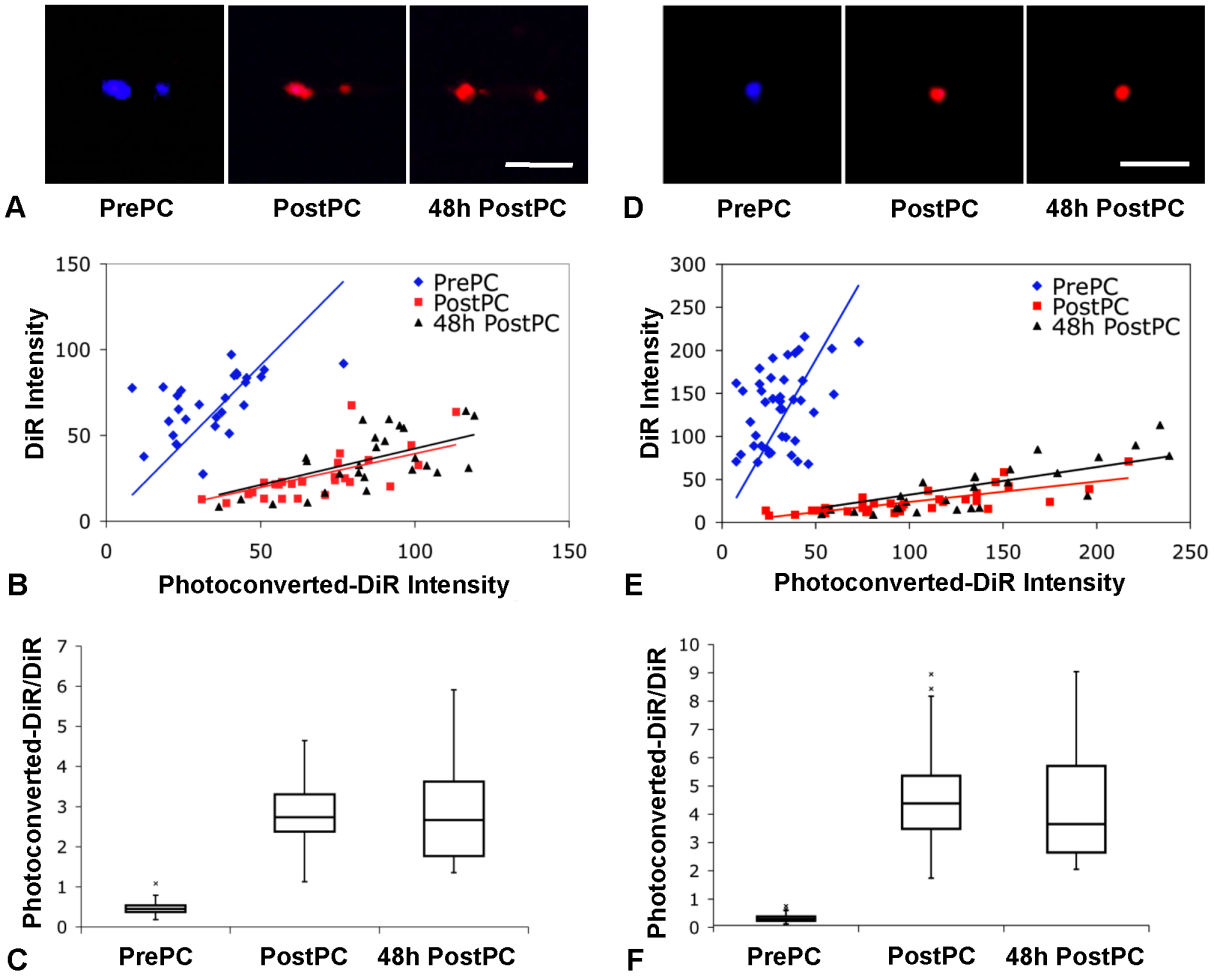
*In vivo* photoconversion of DiR-labeled cells. (A) *In vivo* fluorescence confocal images of DiR-labeled HSPCs acquired before (PrePC), immediately after (PostPC), and 48 h after (48 h PostPC) photoconversion within the skull BM of a live mouse (blue: DiR, >770 nm; red: photoconverted-DiR, 670–720 nm). Scale bar: 50 µm. (B) Plot of fluorescence intensity of *in vivo* HSPCs before (PrePC), immediately after (PostPC), and 48 h after (48 h PostPC) photoconversion for each individual cell. (C) Boxplot of the ratios of the photoconverted-DiR intensity to the DiR intensity, showing ability to photoconvert cells within the skull BM of live mice and to distinguish the change in the fluorescence intensity ratio after photoconversion (p_pre-post_ = 7.72×10^−14^) as well as show the stability of the photoconversion *in vivo* over time (p_post-48hpost_ = 0.82). (D) *In vivo* fluorescence confocal images of DiR-labeled T cells acquired before (PrePC), immediately after (PostPC), and 48 h after (48 h PostPC) photoconversion within the skull BM of a live mouse (blue: DiR, >770 nm; red: photoconverted-DiR, 670–720 nm). Scale bar: 50 µm. (E) Plot of fluorescence intensity of *in vivo* T cells before (PrePC), immediately after (PostPC), and 48 h after (48 h PostPC) photoconversion. (F) Boxplot of the fluorescence intensity ratios showing ability to distinguish the change in fluorescence after photoconversion (p_pre-post_ = 2.59×10^−16^) as well as show the stability of the photoconversion *in vivo* over time (p_post-48hpost_ = 0.94).

**Figure 5:**
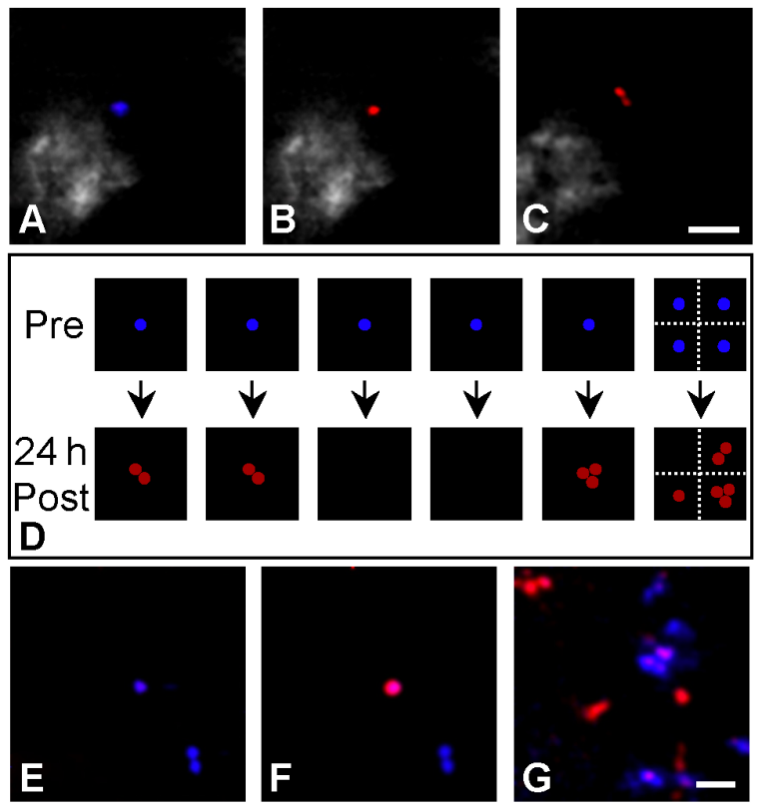
*In vivo* tracking of hematopoietic stem/progenitor cell proliferation. Fluorescence confocal images of DiR-labeled HSPCs acquired in the skull BM of mice (A) before, (B) immediately after, and (C) 24 h after *in vivo* photoconversion (blue: DiR, >770 nm; red: photoconverted-DiR, 670–720 nm). Image (C) shows proliferation of the photoconverted cell. The drawing in figure (D) represents the results of tracking nine cells 24 h after photoconversion in six mice; each square represents one mouse. Series (E)–(G) demonstrates the ability to track HSPCs over long, discontinuous time periods by showing images acquired within the skull bone marrow (E) before, (F) immediately after, and (G) 135 h after *in vivo* photoconversion (blue: DiR, >770 nm; red: photoconverted-DiR, 670–720 nm). Scale bars: 50 µm.

**Figure 6:**
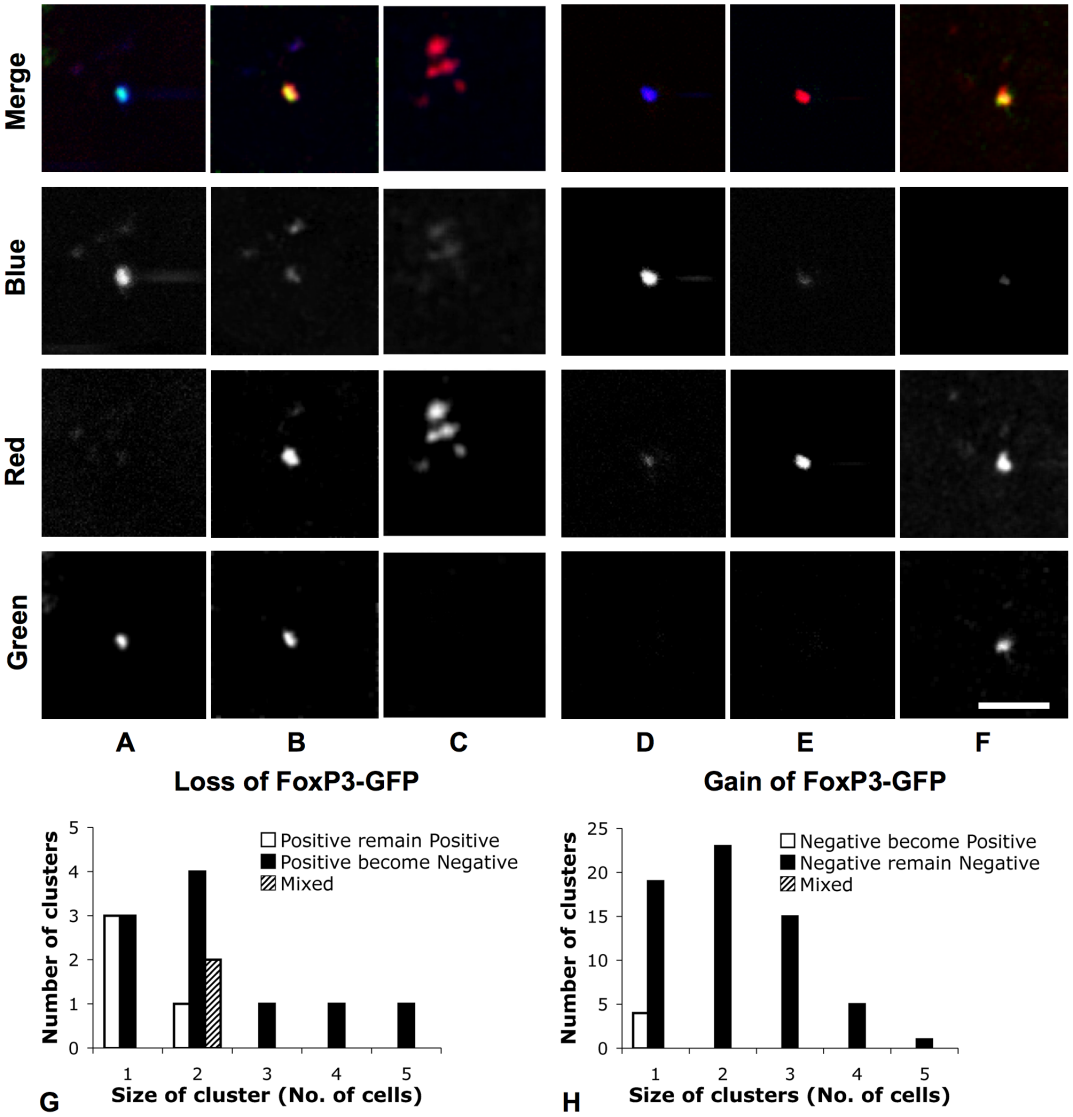
*In vivo* tracking of FoxP3-GFP switching on and off in CD4^+^ T cells. 24 h after adoptive transfer of DiR-labeled FoxP3-GFP positive or negative T cells into RAG2^−/−^ mice, T cells in skull BM were photoconverted and tracked longitudinally (blue: DiR, >770 nm; red: photoconverted-DiR, 670–720 nm; green: GFP, 509–547 nm). Series (A)–(C) shows that 48 h after photoconversion of DiR-labeled FoxP3-GFP-positive T cells (A: before photoconversion, blue+ red− green+; B: after photoconversion, blue− red+ green+), some photoconverted cells (24/30) turned off expression of FoxP3-GFP (C: blue− red+ green−), indicating that non-Tregs can be generated from Tregs in the BM. Series (D)–(F) shows that 48 h after photoconversion of DiR-labeled FoxP3-GFP-negative T cells (D: before photoconversion, blue+ red− green−; E: after photoconversion, blue− red+ green−), a small portion of photoconverted cells (4/113) expressed FoxP3-GFP (F: blue− red+ green+), indicating that FoxP3-GFP Tregs can be generated from FoxP3-GFP-negative T cells in the BM. Scale bar: 50 µm. Charts (G) and (H) show the number of clusters of FoxP3-GFP positive or negative cells 48 h after photoconversion. 20 of 25 FoxP3-GFP-negative cells, which derived from 19 FoxP3-GFP-positive cells, made clusters. All 4 FoxP3-GFP-positive cells derived from 88 FoxP3-GFP-negative cells remained singlets.

Cells were labeled with the fluorescent, lipophilic carbocyanine DiIC_18_(7) (1,1′-dioctadecyl-3,3,3′,3′-tetramethylindotricarbocyanine iodide) (‘DiR’, Invitrogen, Carlsbad, CA). For *in vitro* work (Figs. 2 and 3), cells were labeled for 10–20 min at 37°C at a concentration of 5 µM in RPMI with 0.1% BSA. For the *in vivo* work (Figs. 4, 5, and 6), cells were labeled for 25 min at 37°C at a concentration of 15 µM in RPMI with 1% FBS. Following labeling, cells were washed a minimum of two times to remove unbound dye.

### Bone marrow transplantation

DiR-labeled HSPCs were intravenously injected via the tail vein into 9.5 Gy-irradiated recipient mice.

### Fluorescence spectroscopy

Fluorescence emission spectra of photoconverted and non-photoconverted DiR-labeled cells were acquired using a SPEX FluoroMax-3 (Horiba Scientific, Edison, NJ) at 632 nm excitation with a 3 nm slit width. Emission was collected from 640 nm to 845 nm, using a 5 nm slit width. Excitation spectra of the cells were acquired for 780 nm emission and 670 nm emission, both with a 3 nm slit width. Excitation was collected from 400 nm to 775 nm and from 400 to 665 nm, respectively, both with a 5 nm slit width. The intensities of the emission spectra were then corrected to account for differences in the excitation absorption derived from the excitation spectra.

### *In vivo* confocal imaging

Images were acquired with a non-commercial confocal/multi-photon microscope specifically designed for live animal imaging. The microscope design is described in detail in [45]. Briefly, the system contains three continuous wave lasers, yielding four excitation wavelengths at 491 nm (Dual Calypso, Cobolt AB, Stockholm, Sweden), 532 nm (Dual Calypso, Cobolt AB, Stockholm, Sweden), 561 nm (Jive, Cobolt AB, Stockholm, Sweden), and 635 nm (Radius, Coherent Inc., Santa Clara, CA) The system also contains a Mai Tai femtosecond pulsed laser (Coherent Inc., Santa Clara, CA) for multi-photon and second-harmonic imaging as well as single-photon photoconversion. The scan engine of the system consists of a spinning polygon (Lincoln Laser, Phoenix, AZ) and galvanometer (Cambridge Technology, Bedford, MA), producing images of 500×500 pixels at a frame rate of 30 frames per second. The system contains five photomultiplier tube (PMT) detectors, four for confocal imaging and one for two-photon and second-harmonic imaging. High-resolution images were acquired using a 30× 0.9 NA water-immersion objective (Lomo, St. Petersburg, Russia) which yielded a field of view of 660×660 µm.

*Ex vivo* images were acquired of cells on glass cover slips, and *in vivo* images were acquired of transplanted cells in the skull BM in live mice. Mice were anaesthetized by intraperitoneal injection of a Ketamine/Xylazine cocktail (100 mg/kg Ketamine+15 mg/kg Xylazine) and prepared for *in vivo* imaging by making a small incision in the scalp, creating a skin flap to expose the underlying dorsal surface of the skull. Mice were held in a temperature-controlled tube, positioned on the microscope stage. High-resolution images of cellular details were obtained through the intact mouse skull at depths up to 150 µm from the dorsal surface of the skull. For imaging, DiR was excited using the 635 nm helium-neon laser (Radius). The emitted fluorescence was detected in two channels, either through a 710±50 nm bandpass filter (HHQ710/100M, Chroma Technologies, Rockingham, VT) and 785±25 nm bandpass filter (OS02770, Chroma Technologies, Rockingham, VT) (*set-up A*, Figs. 3A and 3B) or through a 695±27.5 nm bandpass filter (XF3076 695AF55, Omega Optical, Brattleboro, VT) and 770 nm longpass filter (HQ770LP, Chroma Technologies, Rockingham, VT) (*set-up B*, Figs. 3C, 3D, 4, 5, and 6). GFP was excited with the 491 nm solid-state laser (Dual Calypso) and detected through a 528±19 nm bandpass filter (FF01-528/38-25, Semrock, Rochester, NY). Images were captured, after averaging thirty frames, using a Macintosh computer equipped with an Active Silicon snapper card (Active Silicon Chelmsford, MA). Each channel was acquired individually, but simultaneously, in 8-bit grayscale and merged to form an RGB image, using custom developed software (iPhoton). Multiple time points were taken between 1 and 135 hours after cell injection. At the completion of the initial imaging session, the skull was flushed with sterile saline, and the scalp skin flap was closed using 5-0 non-absorbable nylon sutures. For subsequent imaging sessions, the sutures were removed and the area flushed and swabbed with sterile saline.

### Photoconversion

Injected cells were located based on their DiR fluorescence emission. Fluorescence images were acquired before and after photoconversion of the dye for ratiometric imaging. *In vitro* photoconversion was accomplished by illuminating the cell(s) of interest with 8–15 mW of power (scanned over 660×660 µm) at 750 nm (Mai Tai, SpectraPhysics, Newport, Santa Clara, CA) for 5–10 s. *In vivo* photoconversion was accomplished with 32–45 mW of power scanned over 660×660 µm for 20 s. To photoconvert a single cell, a physical mask was placed at an intermediate image plane to restrict the exposure area to the size of a single cell. The ratio of the 685–735 nm (red) fluorescence (*imaging set-up A*, Figs. 3A and 3B) or 668–722 nm (red) fluorescence (*imaging set-up B*, Figs. 3C, 3D, 4, 5, and 6) to the fluorescence greater than 770 nm (near-infrared, NIR) before and after photoconversion was then used to distinguish between photoconverted and non-photoconverted cells within an area of tissue.

### Data analysis

Data analysis was conducted on a cell-by-cell basis. The average intensity of each cell within the blue and the red channels of the image was determined using either ImageJ (NIH, Bethesda, MD) or Matlab (Mathworks, Natick, MA). For the *ex vivo* images and the 1 h time point *in vivo* images, the same region of the images acquired before and after photoconversion was analyzed. The ratio of the intensity in the red channel (685–735 nm for *imaging set-up A* or 668–722 nm for *imaging set-up B*) to the intensity in the blue channel (>770 nm) was calculated for each cell. A two-tailed, two-sample paired t-test with an alpha value of 0.001 was applied to all before (PrePC) - after (PostPC) comparisons. A two-tailed, two-sample unpaired t-test, assuming equal variances (based on evaluation by corresponding f test), with an alpha value of 0.001 was applied to the immediately after (PostPC) - 48 h after (48 h Post PC) comparison. A p-value of less than 0.001 indicated that the samples were statistically different. The boxplots are drawn such that the bottom of the box represents the first quartile (Q1, 25^th^ percentile), the top of the box represents the third quartile (Q3, 75^th^ percentile), the center line represents the median value, and the whiskers extend to the highest and lowest data points or 1.5 times the interquartile range (Q3-Q1), whichever is shorter. Outlier points, those that are greater than 1.5 times the interquartile range, are denoted by x's.

## Results

### DiR photoconversion

Figure 2 shows the excitation and emission spectra of DiR-labeled cells before and after photoconversion. Fluorescence excitation peaks are at 750 nm prior to photoconversion and 650 nm after photoconversion. Illumination at 750 nm is used to induce the photoconversion of the dye. The fluorescence emission spectra, acquired with 632 nm excitation, are shown in Fig. 2B. After photoconversion, the fluorescence emission peak at 780 nm decreased while the peak at 670 nm increased.

Fluorescence confocal images of DiR-labeled HSPCs (Fig. 3A) and T cells (Fig. 3D) acquired *ex vivo* with 635 nm excitation immediately before and after photoconversion show a characteristic change in the fluorescence emission from near-infrared (NIR) (blue channel) to red (red channel) wavelengths following photoconversion, corresponding to the spectral change seen in Fig. 2B. Ratiometric analysis of the images can be used to distinguish photoconverted cells from non-photoconverted cells; non-photoconverted cells will have fluorescence ratio R_b_ (ratio before photoconversion) while photoconverted cells (and their daughter cells) will have fluorescence ratio R_a_ (ratio after photoconversion). A scatter plot of the average fluorescence intensity of each cell in both the blue and red channels of the image, analyzed before and after photoconversion within a single *ex vivo* experiment per cell type, is plotted in Figs. 3B and 3E. From this data, the fluorescence ratio of the intensities in the two channels was calculated for each cell (Figs. 3C and 3F). The average fluorescence ratio of the HSPCs after photoconversion was 12.1 times higher than the ratio prior to photoconversion (R_b_ = 0.53±0.21, R_a_ = 6.42±4.24, p = 8.36×10^−4^). Similarly, the average fluorescence ratio of the T cells after photoconversion was 17.6 times higher than the ratio prior to conversion (R_b_ = 0.36±0.12, R_a_ = 6.35±2.54, p = 1.15×10^−33^), suggesting that good distinction between photoconverted and non-photoconverted cells can be achieved.

### *In vivo* photoconversion of DiR-labeled cells

The ability to distinguish between photoconverted and non-photoconverted cells *in vivo* was then determined. Lethally irradiated C57BL/6 mice were injected with DiR-labeled HSPCs and imaged using IVM one hour after injection. HSPCs were located within the skull BM of live mice based on their DiR fluorescence before photoconversion and were imaged before and after photoconversion as well as 48 h after photoconversion (Fig. 4A). The average fluorescence intensity of each cell, from two *in vivo* experiments per cell type, in both the blue and red channels of the image, is shown in Fig. 4B. After *in vivo* photoconversion, cells yielded an average red-to-NIR fluorescence (red-to-blue channel intensity) ratio 5.7 times higher than the ratio prior to photoconversion (Fig. 4C, R_b_ = 0.52±019, R_a_ = 2.97±0.88, p = 7.72×10^−14^). Figures 4B and 4C also show that the fluorescence ratio is stable 48 h after photoconversion (R_a_ = 2.97±0.88 immediately after and 2.91±1.25 48 h after photoconversion, p = 0.82).

The same ratiometric analysis was repeated for images of injected T cells acquired *in vivo* in the mouse skull BM. Figure 4D shows *in vivo* images of T cells before, immediately after, and 48 hours after photoconversion. Figures 4E and 4F show a similar change in the average fluorescence ratio before and after photoconversion (R_b_ = 0.25±0.13, R_a_ = 4.58±1.68, p = 2.59×10^−16^). They also show that the fluorescence ratio is stable 48 h after photoconversion (R_a_ = 4.58±1.68 immediately after and 4.54±2.30 48 h after photoconversion, p = 0.94), supporting the use of the dye-photoconversion method to track the fate of DiR-labeled cells over extended time periods.

### *In vivo* photoconversion of DiR-labeled HSPCs to track proliferation

Photoconverted cells were then tracked longitudinally, using the photoconversion method and calculated fluorescence ratio to track individual cell division within the BM of live mice. Lethally irradiated C57BL/6 mice were injected with DiR-labeled HSPCs; single cells were targeted and photoconverted one hour after injection. These cells were then imaged again 24 h after photoconversion. In five of the six recipient mice, only one cell within the entire skull BM was photoconverted, to ensure that any photoconverted cells detected at a later time point were progeny from this single converted cell, while in the remaining recipient, four cells were photoconverted at well-separated locations within the BM giving a total of nine cells at the start of the series of experiments. Figures 5A–5C show an example of one of the cells before, immediately after, and 24 h after photoconversion, respectively. Of note, the doublet seen at 24 h (Fig. 5C) must have come from division of the original cell, because only one cell in the entire mouse was photoconverted. Figure 5D shows the outcome of the follow-up imaging of all nine cells at 24 h after photoconversion (each square box denotes a single mouse). From six of the nine photoconverted cells, we observed one singlet, three doublets, and two triplets at the same location in the BM 24 h later, indicating that HSPCs proliferate at the location where they initially home within one hour after intravenous injection.

Interestingly, in the remaining three cases, the photoconverted cell could not be found in the original location or in other regions of the skull BM. It is not likely that the photoconverted HSPCs underwent so many cell divisions within 24 h that the dye was diluted below detection, as we can track cells after at least three cell divisions (see Fig. 6C discussed below). A more plausible explanation is that the HSPCs either migrated deeper into the BM, beyond our 150 µm imaging depth, or migrated out of the skull, possibly by re-entering the circulation. We cannot, however, rule out the possibility that some of the injected cells lost viability and were removed by host cells.

We were also able to perform long-term imaging of HSPCs within the skull bone marrow by tracking photoconverted cells for 135 h (5.6 days). Figures 5E–5G show an example of one cell before, immediately after, and 135 h after photoconversion, respectively, again supporting the use of the dye-photoconversion to track the fate of DiR-labeled cells over extended time periods.

### *In vivo* photoconversion of DiR-labeled T cells to track single cell fate

As a second example of the application of the dye-photoconversion method, we used an additional fluorescent reporter gene that marks cell differentiation or function, thus enabling us to track the *in vivo* differentiation status of individual cells over time. Here we use the technique to track CD4 T cell peripheral differentiation within the BM by examining the gain or loss of their FoxP3 expression. FoxP3 is a transcription factor whose expression in a subset of CD4 T lymphocytes, called regulatory T cells (Tregs), confers immune suppressive function and plays a critical role in maintaining immune self-tolerance [43]. The development of FoxP3-GFP knock-in mice has provided a powerful means to identify the FoxP3-positive Treg cell population [42], [44], but new questions have been raised about the stability of the FoxP3 expression after adoptive transfer [5]–[9]. In standard population-based studies, it has been difficult to exclude the possibility that expansion of a small population of contaminating cells in the starting pool (for example, non-Tregs in a pool of supposedly purified Tregs or vise versa) could be misinterpreted as newly generated cells that lost/gained FoxP3 expression [5]–[8]. Moreover, these population-based studies do not provide information about the cellular location where these transitions take place. To overcome these limitations, we track the gain or loss of FoxP3-GFP expression in CD4 T cells after highlighting them by the *in vivo* dye-photoconversion method. This method enabled unambiguous identification of whether the individual starting CD4 T cells were Tregs or non-Tregs, and provided information on the location where the switching of the phenotype took place *in vivo*.

After isolating FoxP3-positive Tregs from FoxP3-GFP mice, we adoptively transferred DiR-labeled Tregs into RAG2^−/−^ mice. 24 h later, the skull BM of the recipients were imaged, and individual T cells were photoconverted. 48 h after photoconversion, the skull BM was re-imaged to track FoxP3-GFP expression of the photoconverted cells. A total of 19 FoxP3-GFP-positive Tregs were photoconverted, and 48 h later, 32 photoconverted T cells were found in the skull BM cavities. Some photoconverted T cells formed clusters at the same location where a single T cell was previously photoconverted, indicating homeostatic proliferation of CD4 T cells in the BM of lymphopenic mice. Interestingly, 25 of the 32 photoconverted T cells did not express GFP 48 h after photoconversion (Figs. 6A–6C), indicating that some FoxP3-positive Tregs turned off their FoxP3 expression. Additionally, 20 of 25 photoconverted FoxP3-GFP-negative T cells were found in clusters, suggesting that loss of FoxP3 expression in CD4 Tregs in the lymphopenic condition is associated with homeostatic proliferation (Fig. 6G).

We also performed the opposite experiment, starting with FoxP3-GFP-negative CD4 T cells isolated from the FoxP3-GFP mice. In recipients adoptively transferred with only DiR-labeled FoxP3-GFP-negative non-Tregs, 88 FoxP3-GFP-negative T cells were photoconverted, and 48 h later, 139 photoconverted T cells were found in the skull BM cavities, again indicating homeostatic proliferation. Four of the 139 photoconverted T cells expressed GFP, indicating that FoxP3-negative T cells can turn on FoxP3 expression in the BM (Figs. 6D–6F and 6H). These data directly demonstrate the instability of the FoxP3 transcription factor in CD4 T cells in the lymphopenic condition, indicating that Tregs and non-Tregs can switch their phenotypes in the BM.

## Discussion

The DiR dye-photoconversion method uses a commercial lipophilic membrane dye and ratiometric imaging to distinguish photoconverted cells from non-photoconverted cells with high sensitivity. This robust method can be used to highlight and track individual cells to yield temporal and spatial information about the division, differentiation, and movement of cells at the single-cell level. Compared with photoswitchable [18]–[28] and photoconvertible [18], [29]–[40] fluorescent proteins, the NIR photoconvertible membrane dye has a number of advantages: (i) Photoconversion of the DiR dye is permanent. Once photoconverted, the cell does not reacquire the original fluorescence signal. Additionally, the photoconverted fluorescence ratio is stable through cell division and is passed on to the daughter cells so that progeny of a photoconverted cell can be tracked over at least three cell divisions (Fig. 6C). In contrast, for photoconvertible fluorescent proteins, protein turnover leads to a loss of the photoconverted protein and gain of unconverted protein within 6 h of photoconversion [37] and the cell reverts back to the original unconverted color within 24 h of photoconversion in rapidly proliferating cells if no additional photoconversion is performed [41]. This color reversion is a critical obstacle to using photoconvertible fluorescent proteins to track cell division and differentiation. (ii) Any cells, including freshly isolated primary cells, can be labeled with DiR after a short incubation time with no known effect on their homing or proliferation [3]. The dye labeling method avoids gene transfection, which can affect cell differentiation or function. It also enables labeling of cells that cannot be maintained in culture (e.g., HSPCs) or transfected efficiently (e.g., T cells). Although dye transfer to adjacent cells can be a potential source of “environmental contamination” in cell cultures [46], we have not observed evidence of significant dye transfer *in vivo*. (iii) Finally, the third advantage is that the NIR dye can be used with GFP or other fluorescent reporters to track gene expression and cell differentiation.

The change in the fluorescence emission spectrum of the fluorophore used in the DiR dye, 1,1′,3,3,3′,3′-hexamethylindotrycarbocyanine iodide (HITC), has been noted previously by del Monte and Levy [47] and was attributed to chemical degradation. The change in emission after light activation may be due to an irreversible trans-cis photoisomerization of the DiR dye molecule, as described by Mishra, *et al.* [48]. We noted that the photoconversion does not occur when the dye is in solution, but only when it is bound to a cell, further suggesting that conformational change is key to the photoconversion.

Additionally, we noted that cells labeled with a similar dye, DiD (DiIC_18_(5) ‘Vibrant DiD’; Invitrogen, Carlsbad, CA), at a concentration of 5 µM for 15 min could also be photoconverted *in vitro* using 632 nm illumination, changing the detected fluorescence from approximately 670 nm to something near 570 nm. Although the illumination wavelength and intensity were not ideal for using the photoconversion technique *in vivo* with the DiD dye, it suggests that it may be possible to photoconvert the other members of the DiIC_18_ family in addition to DiR.

Using the dye-photoconversion method in combination with IVM, we have shown that HSPCs injected into irradiated recipients divide at the location on the endosteal surface where they initially home within one hour after intravenous injection (Fig. 5). While previous studies have shown that the HSPC microenvironment, or niche, plays an important role in regulating stem cell division and differentiation, those studies did not directly examine the precise location where these events occurred. Previous studies have identified two HSPC niches, one residing near the endosteum [1], [4] and the other separate from osteoblasts and near BM sinusoidal endothelium away from the endosteum (the vascular niche) [2]. It has been suggested that the endosteal niche may favor stem cell quiescence while the vascular niche may favor proliferation, and the HSPCs may shuttle back and forth between the two niches [49]. We have recently shown that the endosteal niche is in fact perivascular [3], so the two niches may have overlapping perivascular components. Here we have further provided evidence that HSPCs do not have to migrate away from the location where they initially home after transplantation in order to proliferate. With additional cell lineage markers, it will be possible to examine if there are specific locations in the BM that support HSPC differentation. It is possible that instead of two separate but static niches, a single niche as a dynamic entity can support HSPC transition through multiple cell states.

Using the same method, we have also shown that homeostatic proliferation of CD4^+^ T cells occurs in the BM (Fig. 6). When combined with a fluorescent reporter gene (FoxP3-GFP), we have shown that CD4^+^ T cells can both acquire and lose FoxP3 expression in the BM after adoptive transfer to lymphopenic mice (Fig. 6). Because we tracked individual cells with a well-defined phenotype, this method avoids the problem of heterogeneity in the starting cell pool that has complicated the interpretation of previous population-based studies [3], [5]–[9]. Additionally, our method allows direct identification of the cellular location where these transitions take place. The finding that Treg cells can lose FoxP3 expression during homeostatic proliferation has been suggested previously [5], [50], [51]. Further studies are needed to determine if this loss is a transient phenomenon and whether the Treg cells will reacquire FoxP3 expression at a later stage, which seems possible given the acquired GFP expression in a subset of GFP-negative CD4-positive transplanted lymphocytes.

In summary, we have developed a simple *in vivo* cell tracking method using DiR dye-photoconversion that can be used with any cell type that can be labeled *ex vivo* and adoptively transferred into recipient animals. Labeling is brief and does not require cell transfection. The NIR-wavelength spectral range of the DiR dye leaves the entire visible spectrum open for use with other fluorescent protein-based reporters that will enable multi-lineage tracking with single-cell resolution longitudinally over time in live animals.
