# Correction: Tracking Single Cells in Live Animals Using a Photoconvertible Near-Infrared Cell Membrane Label

**DOI:** 10.1371/annotation/c05446df-ee55-4072-940c-543adff42086

**Published:** 2013-11-13

**Authors:** Alicia L. Carlson, Joji Fujisaki, Juwell Wu, Judith M. Runnels, Raphaël Turcotte, Joel A. Spencer, Cristina Lo Celso, David T. Scadden, Terry B. Strom, Charles P. Lin

An author was omitted from the article.

Joel A. Spencer was omitted from the author byline; he should be the sixth author and affiliated with the Center for Systems Biology and Wellman Center for Photomedicine, Massachusetts General Hospital and Harvard Medical School, Boston, Massachusetts, United States of America.

The contributions of this author are: contributed reagents/materials/analysis tools. 

